# LncRNA Expression Profiles in Canine Mammary Tumors Identify lnc34977 as a Promoter of Proliferation, Migration and Invasion of Canine Mammary Tumor Cells

**DOI:** 10.3390/vetsci9020082

**Published:** 2022-02-15

**Authors:** Baochun Lu, Juye Wu, Hebi Chen, Shoujun Li, Kun Jia

**Affiliations:** 1 College of Veterinary Medicine, South China Agricultural University, Guangzhou 510642, China; lubaochum@stu.scau.edu.cn (B.L.); wujuye2022@163.com (J.W.); chenhebi@126.com (H.C.); shoujunli@scau.edu.cn (S.L.); 2 Guangdong Provincial Key Laboratory of Prevention and Control for Severe Clinical Animal Diseases, Guangzhou 510642, China; 3 Guangdong Technological Engineering Research Center for Pet, Guangzhou 510642, China

**Keywords:** canine mammary tumor, lncRNA, lncRNA34977, expression profiles

## Abstract

Canine mammary tumor (CMT) is the most common tumor in canines after skin tumors. Long noncoding RNAs (lncRNAs) have crucial roles in human breast tumor initiation and progression, but the role of lncRNAs in canine mammary tumors is unclear. We analyzed the expression profiles of canine mammary tumors and their adjacent non-neoplastic tissue to explore abnormally expressed lncRNAs. LncRNA expression was detected by qRT–PCR. After overexpression of lnc40589 and knockdown of lnc34977 in CMT cells, CCK-8, colony formation, wound healing and Transwell assays were used to assess the proliferation, migration and invasive ability of canine mammary tumor cells. We also established a mammary tumor-bearing nude mouse model. GO analysis and KEGG pathway analysis demonstrated that the differentially expressed lncRNAs were closely related to the mammary tumor. lnc40589 was significantly upregulated and lnc34977 was significantly downregulated in CMTs. In addition, lnc40589 inhibits cell proliferation, migration and invasion, while lnc34977 promotes cell proliferation, migration and invasion. In addition, lnc34977 promotes the development of mammary tumors in animals. Taken together, our study results reveal the lncRNA expression profiles in CMTs and indicate that lnc34977 promotes the development of CMT both in cell culture and in vivo.

## 1. Introduction

Studies have reported that the canine mammary tumor (CMT) is the most common tumor in canines after skin tumors [1]. The incidence of CMT accounts for 50% of all canine tumors [2]. Early detection and treatment of CMT are critical, especially for the malignant subtype.

Currently, X-ray examination, MRI (magnetic resonance imaging) and other modalities to image the tumor from different angles may improve the rate of early diagnosis, but there are still many limitations to these approaches. Importantly, the high proliferation rate and high invasion ability of CMT result in poor prognosis [3,4,5].

In recent years, tumor biomarkers, such as p53, Bcl-2 (B-cell lymphoma-2), Bax and COX-2 (cyclooxygenase-2), have been widely used for early diagnosis, monitoring and determining the prognosis of CMT [6,7,8]; biomarker evaluation has become a conventional detection method, but the sensitivity and specificity of tumor markers for the diagnosis of CMT are limited. Moreover, to better understand and improve the therapeutic effects of treatments, many studies have focused on genome mutations and the transcriptomics of CMT [9,10,11], but there remains no effective tumor biomarker for the early diagnosis of CMT.

Long noncoding RNAs (lncRNAs) are RNA transcripts longer than 200 nucleotides with little protein-coding capacity [12,13,14]. Genome-wide association studies of tumor samples have identified a large number of lncRNAs associated with various types of human cancers. Alterations in lncRNA expression and their mutations promote tumorigenesis and metastasis. LncRNAs hold strong promise as novel biomarkers and therapeutic targets for cancer [15,16,17].

CMT in dogs exhibits a number of clinical and molecular similarities to human breast cancer [18]. However, the performance and potential of lncRNAs in CMT remains unclear.

## 2. Materials and Methods

### 2.1. Sample Collection and Treatment

Thirty tissue samples with mammary tumors and their adjacent non-neoplastic tissue (2 cm from the tumor tissue) were selected from consecutively recruited female dogs undergoing mastectomy at various animal hospitals in Guangzhou, China, between August 2016 and May 2020. These 30 specimens were grade III (G3) poorly differentiated malignant tumors. The specimens were aseptically disassembled, immediately flash frozen in liquid nitrogen and then stored at −80 °C. All canine mammary tissue was diagnosed by pathologists and none had received radiotherapy or chemotherapy.

### 2.2. RNA Extraction and High-Throughput Sequencing

The total RNA was extracted from the samples using TRIzol reagent (TaKaRa, Shiga, Japan) following the manufacturer’s instructions. The total RNA was treated to remove rRNA using Ribo-Zero rRNA Removal Kits (Illumina, San Diego, CA, USA) following the manufacturer’s instructions. RNA libraries were constructed by using rRNA-depleted RNAs with the TruSeq Stranded Total RNA Library Prep Kit (Illumina, San Diego, CA, USA) according to the manufacturer’s instructions. Libraries were evaluated for quality and quantified using the Bioanalyzer 2100 system (Agilent Technologies, Santa Clara, CA, USA). Libraries (10 pM) were denatured as single-stranded DNA molecules, captured on Illumina flow cells, amplified in situ as clusters and finally sequenced for 150 cycles on an Illumina HiSeq Sequencer, according to the manufacturer’s instructions.

### 2.3. Bioinformatics Analysis of Differentially Expressed lncRNAs

Paired-end reads were harvested from an Illumina HiSeq 4000 sequencer and were quality controlled by Q30. After 3′ adaptor trimming and low-quality read removal, cutadapt (NBIS, Sweden) software (v1.9.3) was used for analysis. The high-quality reads were aligned to the dog reference genome (UCSC canFam3) with HISAT2 (CCB, Baltimore, MD, USA) software. Then, guided by the Ensembl gtf gene annotation file, Cuffdiff (Trapnell Lab, St. Louis, MO, USA) software (part of Cufflinks) was used to obtain the expression profiles of lncRNA and mRNA in FPKM, and the fold changes and *p* values were calculated based on FPKM to identify differentially expressed lncRNAs and mRNAs. LncRNA target genes were predicted according to the locations of nearby genes. GO and KEGG pathway analyses were performed on these target genes. GO and KEGG pathway enrichment analyses of the differentially expressed mRNAs were also performed.

### 2.4. Quantitative Real-Time Polymerase Chain Reaction (RT–qPCR) Analysis

Total RNA was reverse-transcribed into complementary DNA (cDNA) using HiScript II Q RT SuperMix for qPCR (Vazyme Biotech, Nanjing, China). Then, an ultraviolet spectrophotometer was used to detect the OD 260/280 value of the RNA specimen to calculate the RNA concentration. Quantitative real-time polymerase chain reaction (RT–qPCR) was performed using ChamQ Universal SYBR qPCR Master Mix (Vazyme Biotech, Nanjing, China). ACTB, as a housekeeping gene, was used as an internal control. The sequences of lncRNAs were determined in Ensemble. The sense and antisense primers encoding lnc40589, lnc34977 and GAPDH mRNA were designed using Primer Premier 6.0 (PREMIER Biosoft, San Francisco, CA, USA) software as follows:

lnc40589 sense primer, 5′-GGAAGGACAGAGCCAACTCC-3′

lnc40589 antisense primer, 5′-AGGCAGCTGCTTGACTGATT-3′

lnc34977 sense primer, 5′-ATTATACCAAGAGTGAGCACCT-3′

lnc34977 antisense primer, 5′-AAAGAGGATTTCTGCCCAT-3′

ACTB sense primer, 5′-GGCATCCTGACCCTGAAGTA-3′

ACTB antisense primer, 5′-GGGGTGTTGAAAGTCTCGAA-3′

Solubility curves were used to evaluate the reliability of the PCR results. The average CT value (amplified power curve inflection point) was taken. Semiquantitative analysis and calculation of the relative expression of the target gene were performed with 2^−ΔΔCt^.

### 2.5. Cells and Culture

CMT cell lines (CHMp and CHMm) in the exponential growth phase were used in this study, both of which were gifted by the Laboratory of Veterinary Surgery, Faculty of Life Sciences, Department of Agriculture, University of Tokyo, Japan. The culture medium was DMEM containing 1% penicillin-streptomycin (Thermo Fisher Scientific, Waltham, MA, USA) and 10% fetal bovine serum (FBS; BI). The incubator (Thermo Fisher Scientific, Waltham, MA, USA) was maintained at 37 °C and 5% CO_2_.

For transfection, 5 × 10^5^ cells were plated on 6-well plates. Plasmid DNA-lipid complexes were prepared according to the protocol and then added to the cells. When transfecting cells with siRNA, the protocol was followed as described for DNA except that P3000 Reagent was not added when diluting the siRNA. SiRNA and negative control (NC) were purchased from Shanghai GenePharma Inc. (Shanghai, China). Construction of overexpression lncRNA40589 plasmid p3FLAG-N-CMV-10-lncRNA40589 utilized p3FLAG-N-CMV-10 vector. The sense and antisense sequences for siRNA were 5′-CCCGGGAUACUGCCUACAUTT-3′ and 5′-AUGUAGGCAGUAUCCCGGGTT-3, respectively. The sense and antisense sequences for siRNA Lnc34977 NC were 5′-UUCUCCGAACGUGUCACGUTT-3′ and 5′-ACGUGACACGUUCGGAGAATT-3′, respectively. To verify that LncRNA34977 and lncRNA40589 affect the function of CHMp cells, lncRNA34977siRNA or NC, p3FLAG-N-CMV-10-lncRNA40589 or p3FLAG-N-CMV-10 were transfected into CHMp cells using Lipofectamine 3000 (Thermo Fisher Scientific, Waltham, MA, USA) and cultured for 24 h.

### 2.6. Cell Counting kit-8 Assay

Cell proliferation was evaluated by a Cell Counting Kit-8 (CCK-8; Beyotime Biotechnology, Shanghai, China) assay according to the manufacturer’s protocol. Briefly, samples of 2000 cells were resuspended and transferred to 96-well plates. Twenty-four hours later, 10 μL CCK-8 was added to the cells and incubated for 3 h. Absorbance was measured at 450 nm by using a Multiskan FC (Thermo Fisher Scientific, Waltham, MA, USA) every 24 h for the next 3 days.

### 2.7. Colony Formation Assay

Aliquots of 200 cells were plated in 6-well plates and cultured for 1–2 weeks. After clones containing ≥20 cells appeared, 4% paraformaldehyde was added for fixation for 20 min, and then the fixed colony was stained with 10% Giemsa dye solution for 20 min. The stained colonies were then photographed and counted. The clone formation rate was calculated as (the clone number/the number of inoculated cells) × 100%.

### 2.8. Wound Healing Assay

The cell layer was grown to 80% confluence and scratched with a 10 μL pipette tip. After washing three times with PBS, the cells were incubated with DMEM without FBS. Wound closure was evaluated at time points of 0 and 24 h. Migration area = blank space area (0 h) − blank space area (24 h).

### 2.9. Cell Invasion Assays

Matrigel was diluted with DMEM at a ratio of 1:8, and then 50 μL of the mixed solution was added to each well to precoat the upper chamber of the Transwell insert (Corning). After incubating at 37 °C and 5% CO_2_ for 4–5 h, 200 μL of cells (1 × 10^5^) was added to the upper chamber of the Transwell insert, and 500 μL of DMEM containing 10% FBS was added to a 24-well plate (Corning) and then cultivated for 24 h. The cells were washed twice with PBS, fixed with 100% methanol and stained with 10% Giemsa for 15 min at room temperature. The uninvaded cells on the chamber membrane were removed with a cotton swab. Cell numbers were counted under a microscope (Leica 200×).

### 2.10. Mammary Tumor-Bearing Nude Mice Model

One hundred microliters of siRNA-lnc34977 CHMp cells (8 × 10^6^) and CHMp cells (8 × 10^6^) in 10% Matrigel basement membrane matrix (Corning) were injected into the penultimate pair of abdominal mammary glands of six 4-week-old female BALB/c nude mice. Mammary glands were injected with siRNA-lnc34977 CHMp cells (8 × 10^6^) for the experimental group and CHMp cells (8 × 10^6^) for the control, and the tumor size and body weight of the mice were measured every 2 days. The mice were sacrificed after 30 days, and the tumor tissue was excised, measured and submitted to pathological examination. All animal procedures were in accordance with animal welfare good practices and rules.

### 2.11. Statistical Analysis

All the measurement data were expressed as mean ± standard deviation, and the results of the fluorescent quantitative PCR were calculated using the 2^−^^ΔΔCt^ method to calculate the relative expression of target genes, and the statistical software SPSS 19.0 (IBM, New York, NY, USA) was used to analyze the calculated results. The remaining statistical analyses were performed between two groups using the *t*-test to compare the differences, and between groups of three or more groups using one-way ANOVA, and the results were statistically significantly different at *p* < 0.05 and highly statistically significantly different at *p* < 0.01. All statistical analyses were performed using GraphPad Prism software version 6.0 (GraphPad Software Inc., San Diego, CA, USA).

## 3. Results

### 3.1. Sample Collection and Identification

Three cases with a pathological diagnosis of canine mammary adenocarcinoma diagnosed by the LABOKLIN Animal Clinical Laboratory (Germany) were used for Whole Genome Sequencing as three replicates. Tumor tissues (B1, D1 and G1) were used as the experimental group, and adjacent non-neoplastic tissue (B2, D2 and G2) were used as the control group.

### 3.2. lncRNA and mRNA Profiles

**Figure 1:**
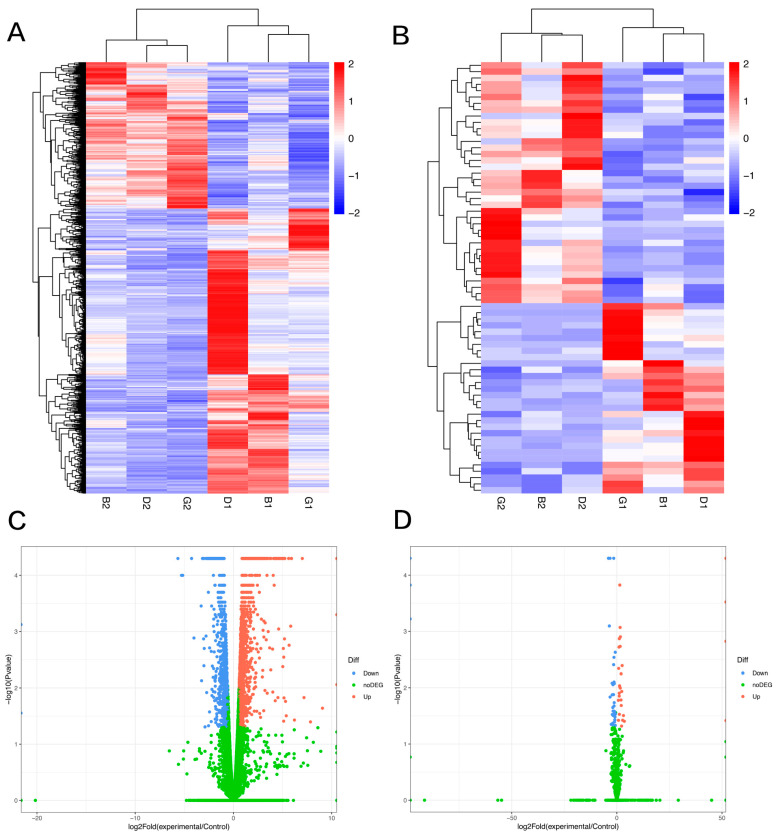
Differentially expressed gene analysis from RNA-seq (fold change > 1.5, *p* < 0.05). (**A**,**B**) Heatmap plot of mRNA and lncRNA. Red indicates high relative expression; blue indicates low relative expression. (**C**,**D**) Volcano plots of mRNA and lncRNA expression.

Overall, 2208 significantly differentially expressed mRNAs and 68 significantly differentially expressed lncRNAs were identified in canine mammary tumor tissues compared with adjacent non-neoplastic tissue. Among them, 1459 mRNAs and 64 lncRNAs were upregulated, and 749 mRNAs and 38 lncRNAs were downregulated. Hierarchical cluster analysis was used to investigate the expression levels of the mRNAs (Figure 1A) and lncRNAs (Figure 1B). The mRNAs and lncRNAs with statistically significant differential expression between groups were plotted in volcano plots, as shown in Figure 1C,D.

### 3.3. Differentially Expressed Gene Analysis from a Public Database

**Figure 2:**
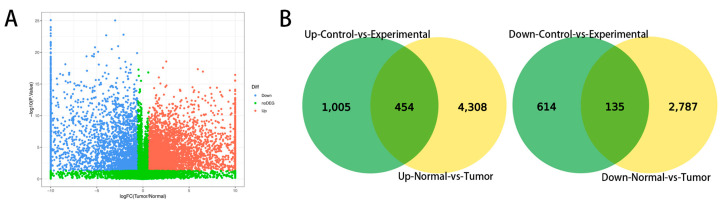
Differentially expressed gene analysis from a public database. (fold change > 1.5, *p* < 0.05). (**A**) Volcano plots of gene expression. (**B**) Venn diagram of common differentially expressed genes. Green indicates data generated in this study and yellow indicates publicly available data.

Canine mammary tumor data (GSE119810) were downloaded from the public database GEO. This dataset included 158 cancer samples and 64 adjacent normal tissue samples. Overall, 7684 significantly differentially expressed genes were identified and plotted in volcano plots, as shown in Figure 2A. Among them, 2922 genes were upregulated and 4762 genes were downregulated. Common differential gene analysis was performed between the public database and our data, and a Venn diagram was drawn. In total, there were 454 upregulated genes and 135 downregulated genes common to both datasets Figure 2B. Tumors were found to have more highly expressed genes than adjacent non-neoplastic tissue.

### 3.4. GO and KEGG Pathway Analysis of Common Differentially Expressed Genes

**Figure 3:**
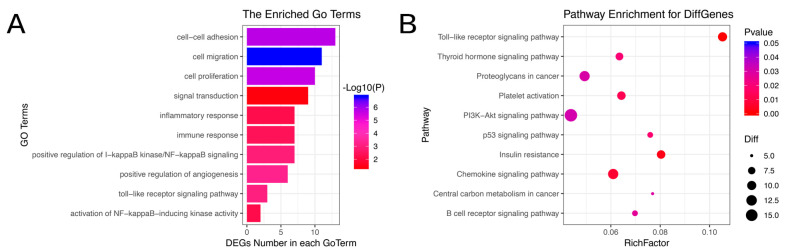
GO and KEGG pathway analysis of common differentially expressed genes. (**A**) GO analysis of common differentially expressed genes. (**B**) KEGG pathway analysis of common differentially expressed genes.

We annotated the transcripts for GO analysis (Figure 3A) and KEGG pathway analysis. The GO analysis demonstrated that most genes were closely related to the mammary tumor, including angiogenesis, cell proliferation, inflammatory response and NIK/NF-kappaB signaling. The KEGG pathway analysis demonstrated that differentially expressed genes were enriched in the Toll−like receptor signaling pathway, PI3K−Akt signaling pathway and p53 signaling pathway (Figure 3B).

### 3.5. lncRNA–miRNA–mRNA Network

**Figure 4:**
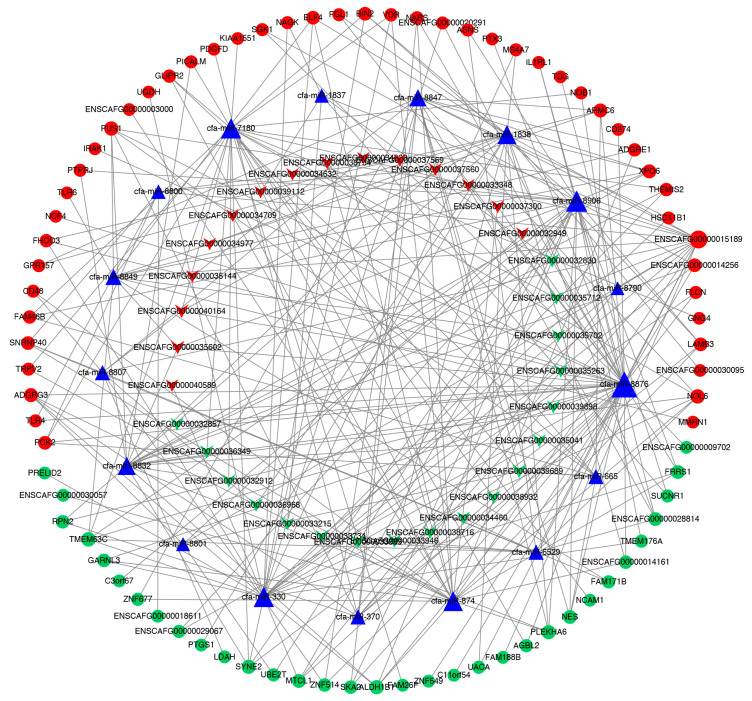
lncRNA–miRNA–mRNA network. Triangles indicate miRNAs, V-shapes indicate lncRNAs and round shapes indicate mRNAs. Red indicates upregulated genes; green indicates downregulated genes.

We used MiRanda software (New York, NY, USA) to predict the miRNA–lncRNA targeting relationship and miRNA–mRNA targeting relationship of the differentially expressed lncRNAs. A total of 9639 pairs were screened, involving 63 differentially expressed lncRNAs, 94 differentially expressed mRNAs, 239 not differentially expressed mRNAs and 176 not differentially expressed miRNAs. We used Cytoscape software (https://cytoscape.org/, accessed on 1 January 2022) to display the relationships in a network diagram. To further obtain reliable candidate genes, a subnetwork was screened from the network diagram. The mRNAs and lncRNAs in the network are significantly differentially expressed and can be targeted by the same miRNA at the same time (Figure 4).

### 3.6. Expression of Differential lncRNAs Verified by RT-qPCR

**Figure 5:**
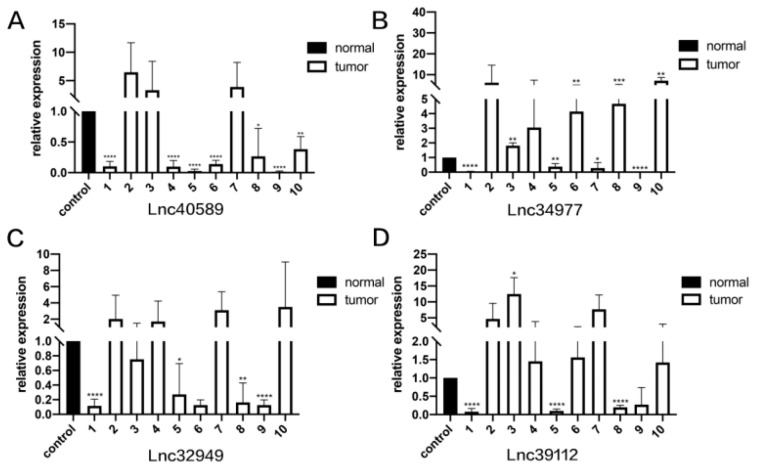
The expression levels of lncRNAs in CMT and adjacent non-neoplastic tissue measured by qRT–PCR. Samples 1–10. (**A**) The expression levels of lncRNA40589 in CMT and adjacent non-neoplastic tissue. (**B**) The expression levels of lncRNA34977 in CMT and adjacent non-neoplastic tissue. (**C**) The expression levels of lncRNA32949 in CMT and adjacent non-neoplastic tissue. (**D**) The expression levels of lncRNA39112 in CMT and adjacent non-neoplastic tissue. (* *p* < 0.05, ** *p* < 0.01, *** *p* < 0.001 and **** *p* < 0.0001).

**Figure 6:**
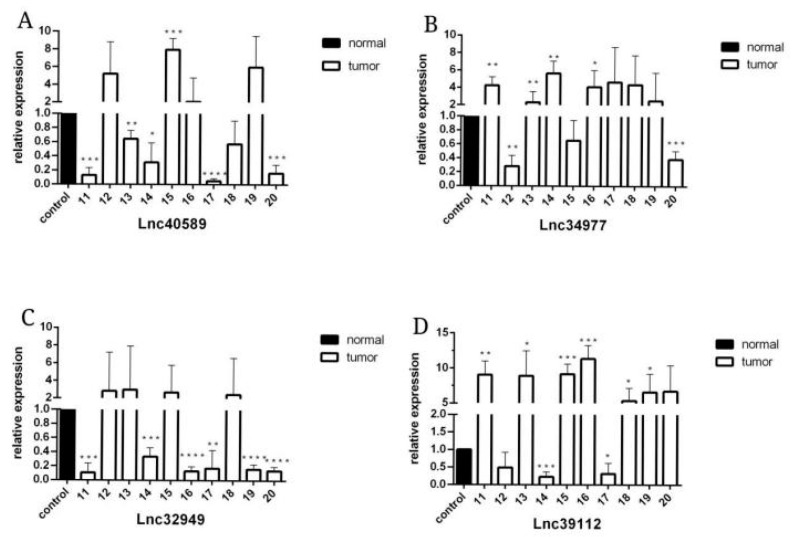
The expression level of lncRNAs in CMT and adjacent non-neoplastic tissue measured by qRT–PCR. Samples 11–20. (**A**) The expression levels of lncRNA40589 in CMT and adjacent non-neoplastic tissue. (**B**) The expression levels of lncRNA34977 in CMT and adjacent non-neoplastic tissue. (**C**) The expression levels of lncRNA32949 in CMT and adjacent non-neoplastic tissue. (**D**) The expression levels of lncRNA39112 in CMT and adjacent non-neoplastic tissue. (* *p* < 0.05, ** *p* < 0.01, *** *p* < 0.001 and **** *p* < 0.0001).

**Figure 7:**
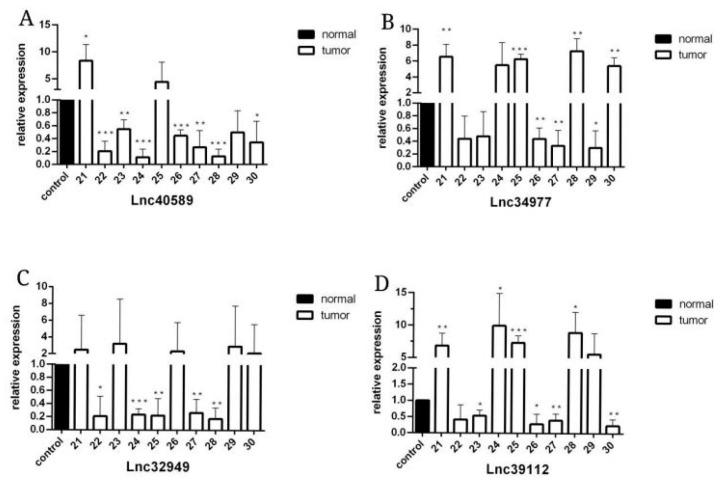
The expression level of lncRNAs in CMT and adjacent non-neoplastic tissue measured by qRT–PCR. Samples 21–30. (**A**) The expression levels of lncRNA40589 in CMT and adjacent non-neoplastic tissue. (**B**) The expression levels of lncRNA34977 in CMT and adjacent non-neoplastic tissue. (**C**) The expression levels of lncRNA32949 in CMT and adjacent non-neoplastic tissue. (**D**) The expression levels of lncRNA39112 in CMT and adjacent non-neoplastic tissue. (* *p* < 0.05, ** *p* < 0.01, *** *p* < 0.001).

To verify the reliability of the sequencing results, we selected four upregulated lncRNAs with obvious differences and statistical significance for qRT–PCR verification. The results (Figure 5, Figure 6 and Figure 7) demonstrated that in canine mammary tumors lncRNA34977 demonstrated high expression, while the expression of lncRNA40589, lncRNA32949 and lncRNA39112 was low. The results of lnc34977 are consistent with the sequencing results, and the results of lnc40589 are opposite to the sequencing results, but the difference is obvious. Therefore, lnc34977 and lnc40589 were selected for follow-up experiments.

### 3.7. Overexpression of lnc40589 and Knockdown of lnc34977 Inhibited the Proliferation of CMT Cells

**Figure 8:**
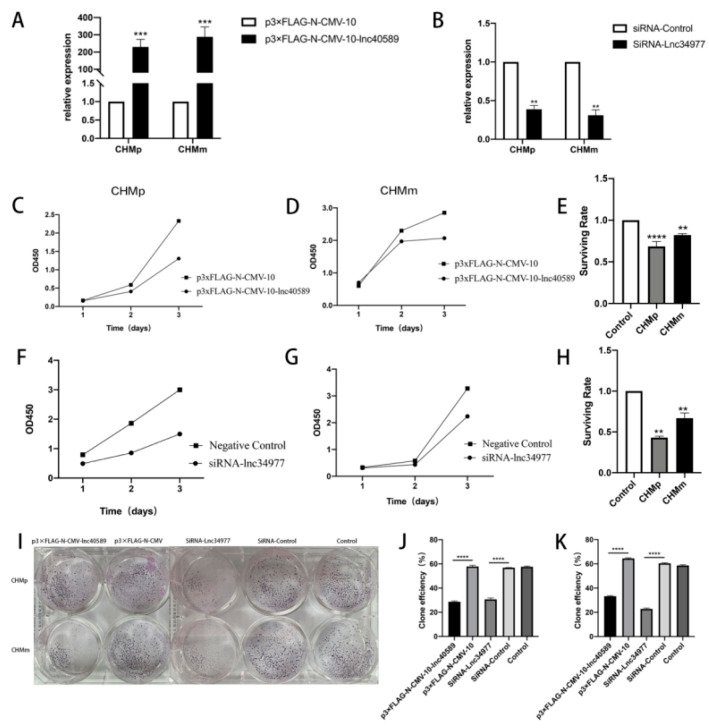
Overexpression of lnc40589 and knockdown of lnc34977 inhibited the proliferation of CMT cells. (**A**,**B**) Relative expression of lnc40589 and lnc34977 after transfection was detected by qRT–PCR. (**C**,**D**) Growth curve of CHMp and CHMm after overexpressing lnc40589. (**E**) Survival rate of CHMp and CHMm after overexpression of lnc40589. (**F**,**G**) Growth curve of CHMp and CHMm after knocking down lnc34977. (**H**) Survival rate of CHMp and CHMm after knockdown of lnc34977. (**I**) Clone formation assay. (**J**,**K**) Clone efficiency of CHMp and CHMm. Three intra-group replicates were performed in each experimental subgroup. (** *p* < 0.01, *** *p* < 0.001 and **** *p* < 0.0001).

To further explore the function of lnc40589 and lnc34977 in the progression of canine mammary tumors, we overexpressed lnc40589 in CHMp and CHMm cells by constructing an overexpression plasmid (p3×FLAG-N-CMV-lnc40589) and knocked down lnc34977 in CHMp and CHMm cells using siRNA. p3×FLAG-N-CMV-lnc40589 effectively overexpressed lnc40589, and siRNA-lnc34977 effectively silenced lnc34977 expression (Figure 8A,B). The CCK-8 assay (Figure 8C–H) and colony formation assay (Figure 8I–K) were used to measure cell proliferation. OD values at 0, 24 and 48 h were used to draw the growth curve. Survival rates were calculated using the OD values measured at 48 h. The assay demonstrated that after overexpressing lnc40589 and silencing lnc34977, the cell proliferation ability of CHMp and CHMm cells was suppressed compared to that of the control group (*p* < 0.05). After overexpressing lnc40589, the survival rate was reduced to 68.51% in CHMp and 82.23% in CHMm. After silencing lnc34977, the survival rate was reduced to 43.03% in CHMp and 66.78% in CHMm. Thus, the colony formation was weakened.

### 3.8. Overexpression of lnc40589 and Knockdown of lnc34977 Inhibited the Migration of CMT Cells

**Figure 9:**
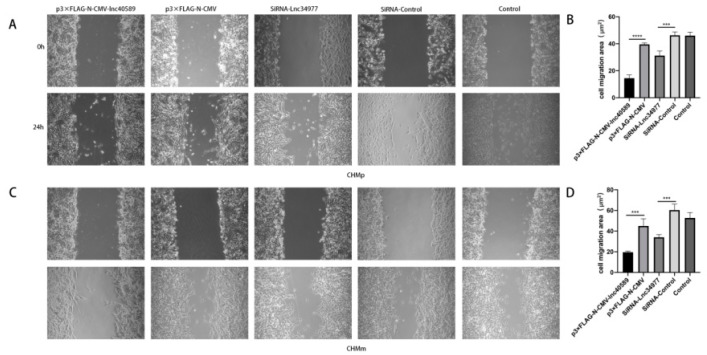
Overexpression of lnc40589 and knockdown of lnc34977 inhibited the migration of CMT cells. (**A**) CHMp migration observed under a microscope before and after overexpressing lnc40589 and knocking down lnc34977. (**B**) Comparison of cell migration areas of CHMp. (**C**) CHMm migration observed under the microscope before and after overexpressing lnc40589 and knocking down lnc34977. (**D**) Comparison of cell migration areas of CHMp. Three intra-group replicates were performed in each experimental subgroup. (*** *p* < 0.001 and **** *p* < 0.0001).

The wound-healing assay demonstrated that after 24 h, the wounds in the experimental groups healed more slowly and the area of cell migration was reduced in both CHMp and CHMm cells (*p* < 0.001) compared with the control groups and NC group. There was no significant difference between the control groups and the NC group (*p* > 0.05) (Figure 9).

### 3.9. Overexpression of lnc40589 and Knockdown of lnc34977 Inhibited Invasion of CMT Cells

**Figure 10:**
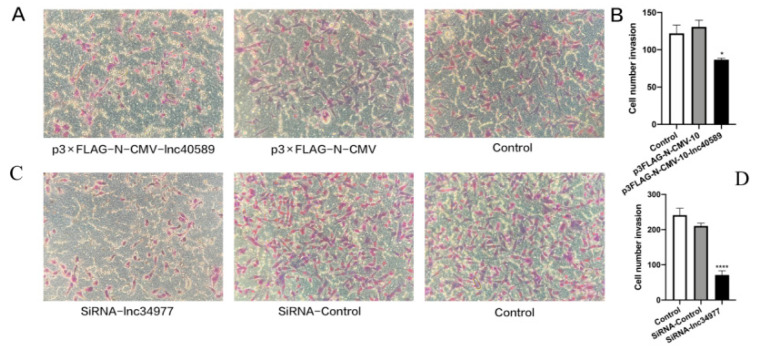
Overexpression of lnc40589 and knockdown of lnc34977 inhibited the invasion of CMT cells. (**A**,**C**) CHMp and CHMm invasion detected by Transwell assay. (**B**,**D**) Numbers of invading CHMp and CHMm cells. Three intra-group replicates were performed in each experimental subgroup. (* *p* < 0.05 and **** *p* < 0.0001).

Transwell assays demonstrated that after overexpressing lnc40589 and silencing lnc34977, the invasion abilities of CHMp and CHMm were decreased compared with those of the control groups and NC (*p* < 0.05). There was no significant difference between the control groups and the NC group (*p* > 0.05) (Figure 10).

### 3.10. Knockdown of lnc34977 in a Mammary Tumor-Bearing Nude Mouse Model

**Figure 11:**
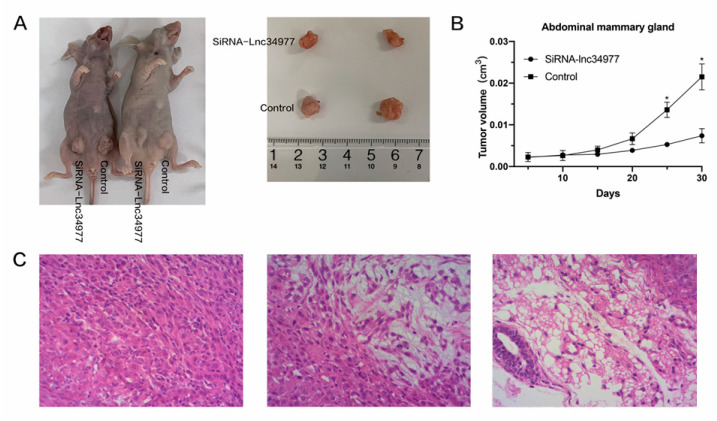
Knockdown of lnc34977 inhibited mammary tumors in a nude mouse model. (**A**) Representative images of tumor-bearing mice. (**B**) The tumor volume growth curves after injections. (**C**) Representative images of hematoxylin and eosin (HE) staining of tumor samples, in which fusiform pleomorphic tumor cell proliferation infiltration is observed in all three images. (* *p* < 0.05).

After 30 days, the tumor size in the siRNA-lnc34977 group was smaller than that in the NC group. The tumors were irregularly spherical, with an uneven surface (not smooth), a nodular feel and a firm texture, and their clinical features were consistent with mammary tumor (Figure 11A,B). The tumor samples were stained with hematoxylin and eosin (HE) (Figure 11C), and pathological analysis of the samples, which showed proliferative infiltration of polyhedral to fusiform pleomorphic mammary tumor cells. The in vitro experiments demonstrated that knockdown of lnc34977 inhibited the development of mammary tumors in animals.

## 4. Discussion

In the diagnosis and treatment of canine mammary tumors, most canine malignant mammary tumors are already advanced at the time of diagnosis [19,20] and treatment options are extremely limited. Therefore, novel therapeutic strategies with better efficacy or fewer side effects are urgently needed. Emerging evidence suggests that lncRNAs play a key role as oncogenes or tumor suppressors in the regulation of many human cancer types [21,22,23,24,25], including breast tumors. The aberrant and differential expression of lncRNAs in breast tumors has been frequently reported [26]. In our study, we used high-throughput sequencing to analyze CMT tissues and adjacent non-tumor tissues to explore the expression profiles of lncRNAs and mRNAs. Our work demonstrated some lncRNAs differentially expressed in CMT, which point to the research potential of lncRNAs in CMT, and enrich the research basis of CMT.

In human medical studies, the effect of lncRNAs on the tumorigenic development of breast tumors was reported. Yan-Xing Guan et al. highlighted that lncRNA SNHG20 regulates HER2 through miR-495 and promotes proliferation, invasion and migration of breast tumor cells [27]. Wenyan Zhao et al. demonstrated that lncRNA HOTAIR promotes proliferation, invasion and migration of breast tumor cells through the miR- 20a-5p/HMGA2 axis, affecting cell growth, metastasis and apoptosis of breast tumors [28]. Kim et al. demonstrated that lncRNA MALAT1 inhibited breast tumor metastasis [29]. However, to date, there are no reports on the aberrant expression of lncRNAs in CMTs.

At present, high-throughput sequencing has become one of the more important ways to analyze lncRNA relationship networks, and more and more lncRNAs are explored by applying this technique [30]. In this study, we used high-throughput sequencing technology to analyze CMT tissues and adjacent non-tumor tissues to explore the expression profiles of lncRNAs and mRNAs. To verify the reliability of the sequencing, we screened two lncRNAs with differential expression, namely lncRNA34977 and lncRNA40589. Many lncRNAs act as promoters or suppressors in tumors, for example, LncRNA-42060 regulates the miR-204-5p/SOX4 axis in canine mammary tumor cells by regulating tumor development [31], and in this process, lncRNA-42060 promotes the development of canine mammary tumors. Similarly, we found that lncRNA34977 promoted proliferation, migration and invasion of canine mammary tumor cells; we also observed that lncRNA40589 inhibited proliferation, migration and invasion of canine mammary tumors, whereas lnc40589 overexpression and lnc34977 knockdown inhibited proliferation, migration and invasion of CHMp and CHMm cells. The results of the cell culture experiments were consistent with the results of qRT-PCR, but differed from the sequencing results, which may be due to the small number of samples sequenced. To further confirm the effect of lncRNA34977 on canine mammary tumors, we constructed a nude mouse model of mammary tumors, and the knockdown of lncRNA34977 suppressed mammary tumorigenesis in the animals.

Increasingly, lncRNAs have been demonstrated to be aberrantly expressed in a variety of human cancers. However, for other animals, lncRNAs have not been verified to be aberrantly expressed. As a companion to humans, the treatment of various diseases in dogs has become extra important, and we should be constantly searching for novel therapeutic modalities for the treatment of canine mammary tumors, which have unlimited possibilities with lncRNAs evidenced as new targets for tumors. Our work fills the research gap of lncRNAs in canine mammary tumors, which has important implications for later in-depth studies of the molecular mechanisms of CMT.

Overall, this study represents a comprehensive analysis of the expression profiles of lncRNAs and mRNAs in CMT tissues and adjacent non-tumor tissues using high-throughput sequencing, and it identified lncRNA40589 and lncRNA34977 as being differentially expressed. Our results demonstrated that lnc40589 inhibited the proliferation, migration and invasion of CMT cells, whereas lncRNA34977 promoted the proliferation, migration and invasion of CMT cells and promoted mammary tumorigenesis in mice.

## 5. Conclusions

Taken together, our findings reveal the lncRNA expression profiles in canine mammary tumors and indicate that lnc34977 promotes canine mammary tumors in cultured cells.

## Data Availability

The datasets for this study can be found in the NCBI’s Gene Expression Omnibus and are accessible through GEO Series accession number GSE137825 (https://www.ncbi.nlm.nih.gov/geo/query/acc.cgi?acc=GSE137825, accessed on 29 November 2021).

## References

[1] Benjamin S.A., Lee A.C., Saunders W.J. (1999). Classification and behavior of canine mammary epithelial neoplasms based on life-span observations in beagles. Vet. Pathol..

[2] Nakagawa T., Uyama R., Ohashi E., Takahashi T., Hong S.H., Mochizuki M., Matsunaga S., Nishimura R., Sasaki N. (2002). The expression of sialyl Lewis X in canine and feline mammary gland tumors. J. Vet. Med. Sci..

[3] Iturriaga M.P., Paredes R., Arias J.I., Torres C.G. (2017). Meloxicam decreases the migration and invasion of CF41.Mg canine mammary carcinoma cells. Oncol. Lett..

[4] Elshafae S.M., Hassan B.B., Supsavhad W., Dirksen W.P., Camiener R.Y., Ding H., Tweedle M.F., Rosol T.J. (2016). Gastrin-releasing peptide receptor (GRPr) promotes EMT, growth, and invasion in canine prostate cancer. Prostate.

[5] Diessler M.E., Castellano M.C., Portiansky E.L., Burns S., Idiart J.R. (2017). Canine mammary carcinomas: Influence of histological grade, vascular invasion, proliferation, microvessel density and VEGFR2 expression on lymph node status and survival time. Vet. Comp. Oncol..

[6] Pennings J.L.A., Van Dycke K.C.G., van Oostrom C.T.M., Kuiper R.V., Rodenburg W., de Vries A. (2012). Biomarker discovery using a comparative omics approach in a mouse model developing heterogeneous mammary cancer subtypes. Proteomics.

[7] Carvalho M.I., Pires I., Prada J., Raposo T.P., Gregorio H., Lobo L., Queiroga F.L. (2017). High COX-2 expression is associated with increased angiogenesis, proliferation and tumoural inflammatory infiltrate in canine malignant mammary tumours: A multivariate survival study. Vet. Comp. Oncol..

[8] Anadol E., Yar S.A., Gultiken N., Karakas K., Alcigir E., Alkan H., Kanca H. (2017). Expression of iNOS, COX-2 and VEGF in canine mammary tumours and non-neoplastic mammary glands: Association with clinicopathological features and tumour grade. Acta Vet. Hung..

[9] Yoshikawa Y., Morimatsu M., Ochiai K., Ishiguro-Oonuma T., Wada S., Orino K., Watanabe K. (2015). Reduced canine BRCA2 expression levels in mammary gland tumors. BMC Vet. Res..

[10] Surdyka M., Slaska B. (2017). Defect in ND2, COX2, ATP6 and COX3 mitochondrial genes as a risk factor for canine mammary tumour. Vet. Comp. Oncol..

[11] Qiu H.B., Sun W.D., Yang X., Jiang Q.Y., Chen S., Lin D.G. (2015). Promoter mutation and reduced expression of BRCA1 in canine mammary tumors. Res. Vet. Sci..

[12] Yang W., Qian Y., Gao K., Zheng W., Wu G., He Q., Chen Q., Song Y., Wang L., Wang Y. (2021). LncRNA BRCAT54 inhibits the tumorigenesis of non-small cell lung cancer by binding to RPS9 to transcriptionally regulate JAK-STAT and calcium pathway genes. Carcinogenesis.

[13] Luo R., Li L., Hu Y.X., Xiao F. (2021). LncRNA H19 inhibits high glucose-induced inflammatory responses of human retinal epithelial cells by targeting miR-19b to increase SIRT1 expression. Kaohsiung J. Med. Sci..

[14] Bertone P., Stolc V., Royce T.E., Rozowsky J.S., Urban A.E., Zhu X., Rinn J.L., Tongprasit W., Samanta M., Weissman S. (2004). Global identification of human transcribed sequences with genome tiling arrays. Science.

[15] Xing C., Sun S.G., Yue Z.Q., Bai F. (2021). Role of lncRNA LUCAT1 in cancer. Biomed. Pharm..

[16] Peng W.X., Koirala P., Mo Y.Y. (2017). LncRNA-mediated regulation of cell signaling in cancer. Oncogene.

[17] Luo Y., Wang Q., Teng L., Zhang J., Song J., Bo W., Liu D., He Y., Tan A. (2020). LncRNA DANCR promotes proliferation and metastasis in pancreatic cancer by regulating miRNA-33b. FEBS Open Bio.

[18] Burrai G.P., Tanca A., De Miglio M.R., Abbondio M., Pisanu S., Polinas M., Pirino S., Mohammed S.I., Uzzau S., Addis M.F. (2015). Investigation of HER2 expression in canine mammary tumors by antibody-based, transcriptomic and mass spectrometry analysis: Is the dog a suitable animal model for human breast cancer?. Tumour. Biol..

[19] Sleeckx N., de Rooster H., Veldhuis K.E., Van Ginneken C., Van Brantegem L. (2011). Canine mammary tumours, an overview. Reprod. Domest. Anim..

[20] Betz D., Schoenrock D., Mischke R., Baumgartner W., Nolte I. (2012). Postoperative treatment outcome in canine mammary tumors. Multivariate analysis of the prognostic value of pre- and postoperatively available information. Tierarztl Prax Ausg K Kleintiere Heimtiere.

[21] Wu D., Zhu J., Fu Y., Li C., Wu B. (2021). LncRNA HOTAIR promotes breast cancer progression through regulating the miR-129-5p/FZD7 axis. Cancer Biomark..

[22] Chen G.Q., Liao Z.M., Liu J., Li F., Huang D., Zhou Y.D. (2021). LncRNA FTX Promotes Colorectal Cancer Cells Migration and Invasion by miRNA-590-5p/RBPJ Axis. Biochem. Genet..

[23] Zhang X.J., Qi G.T., Zhang X.M., Wang L., Li F.F. (2021). lncRNA RHPN1-AS1 promotes the progression of endometrial cancer through the activation of ERK/MAPK pathway. J. Obstet. Gynaecol. Res..

[24] Zhuang C., Ma Q., Zhuang C., Ye J., Zhang F., Gui Y. (2019). LncRNA GClnc1 promotes proliferation and invasion of bladder cancer through activation of MYC. FASEB J..

[25] Xu J., Zhang J. (2020). LncRNA TP73-AS1 is a novel regulator in cervical cancer via miR-329-3p/ARF1 axis. J. Cell Biochem..

[26] Wang G., Liu C., Deng S., Zhao Q., Li T., Qiao S., Shen L., Zhang Y., Lu J., Meng L. (2016). Long noncoding RNAs in regulation of human breast cancer. Brief Funct. Genom..

[27] Guan Y.X., Zhang M.Z., Chen X.Z., Zhang Q., Liu S.Z., Zhang Y.L. (2018). Lnc RNA SNHG20 participated in proliferation, invasion, and migration of breast cancer cells via miR-495. J. Cell Biochem..

[28] Zhao W., Geng D., Li S., Chen Z., Sun M. (2018). LncRNA HOTAIR influences cell growth, migration, invasion, and apoptosis via the miR-20a-5p/HMGA2 axis in breast cancer. Cancer Med..

[29] Kim J., Piao H.L., Kim B.J., Yao F., Han Z., Wang Y., Xiao Z., Siverly A.N., Lawhon S.E., Ton B.N. (2018). Long noncoding RNA MALAT1 suppresses breast cancer metastasis. Nat. Genet..

[30] Qian X., Zhao J., Yeung P.Y., Zhang Q.C., Kwok C.K. (2019). Revealing lncRNA Structures and Interactions by Sequencing-Based Approaches. Trends Biochip. Sci..

[31] Xu E., Hu M., Ge R., Tong D., Fan Y., Ren X., Liu Y. (2021). LncRNA-42060 Regulates Tamoxifen Sensitivity and Tumor Development via Regulating the miR-204-5p/SOX4 Axis in Canine Mammary Gland Tumor Cells. Front. Vet. Sci..

